# Gene–diet-related factors of hyperglycaemia in postmenopausal women

**DOI:** 10.1007/s13353-018-0434-9

**Published:** 2018-02-20

**Authors:** Bogna Grygiel-Górniak, Elżbieta Kaczmarek, Maria Mosor, Juliusz Przysławski, Jerzy Nowak

**Affiliations:** 1 Department of Rheumatology and Internal Diseases, Poznan University of Medical Sciences, 28 Czerwca Street 135/147, 61545 Poznan, Poland; 2 Department of Bioinformatics and Computational Biology, Poznan University of Medical Sciences, Poznan, Poland; 3 Institute of Human Genetics, Polish Academy of Sciences, Poznań, Poland; 4 Department of Bromatology and Human Nutrition, Poznan University of Medical Sciences, Poznan, Poland

**Keywords:** Newly diagnosed hyperglycaemia, Postmenopausal obesity, Genetic background, Nutritional habits

## Abstract

As ageing and increased body fat are the signs of insulin resistance, we have studied whether the presence of Pro12Ala and C1431T of peroxisome proliferator-activated receptor gamma 2 gene and Trp64Arg of beta 3-adrenergic receptor gene may predispose to the hyperglycaemia development in postmenopausal women, who have never undergone hypoglycaemic treatment. The distributions of selected allele and genotype frequencies were determined by the PCR–RFLP method in normo- and hyperglycaemic, who have never been diagnosed and treated for diabetes mellitus were measured. The amount of body fat and lean body mass (LBM) were assessed by the bioimpedance method and nutritional habits by 7-day dietary recall. There were no differences between the distribution of genotypes and the allele frequencies of the Pro12Ala, C1431T and Trp64Arg polymorphisms in normo- and hyperglycaemic women. Hyperglycaemic women were characterized by visceral obesity, hypertension, higher serum insulin and triglycerides, higher intake of fat and lower consumption of complex carbohydrates and B vitamins. Normoglycaemic women with Pro12Pro polymorphism acquired higher energy from dietary fat (*p* < 0.0276) and lower energy from carbohydrates (*p* < 0.0480) than normoglycaemic Ala12 carriers. Subjects with Pro12Pro polymorphism and LBM > 58% of total body mass or with Trp64Trp and normal triglycerides have higher chance of normoglycaemia. Genotyping for Pro12Ala and Trp64Arg polymorphism in postmenopausal women may have the clinical benefit of predicting hyperglycaemia, thereby contributing to the prevention of diabetes mellitus development in the future. However, not only the genetic background but also the dietary habits (intake of fat, carbohydrates and B vitamins) determine the risk of hyperglycaemia.

## Introduction

The influence of genetic and nutritional factors is underlined in the development of hyperglycaemia. Many researchers clearly indicate the role of peroxisome proliferator-activated receptor gamma 2 (*PPARγ2*) gene (polymorphisms: Pro12- ?>Alars1801282 and C1431Trs3856806) and beta-3 adrenergic receptor (*ADRβ3*) gene (polymorphism: Trp64Argrs4994) among the candidate genes for hyperglycaemia and development of type 2 diabetes mellitus (Altshuler et al. 2000; Bell and Polonsky 2001; Grygiel-Górniak 2014). Pro12Pro polymorphism seems to predispose to diabetes mellitus, whereas the presence of *Ala* allele shows “the protective role” of glycaemic complications (Altshuler et al. 2000). The study of 3914 French Caucasians shows that the 6-year risk of hyperglycaemia was lower in Ala12 carriers than in Pro12Pro subjects (Jaziri et al. 2006). In addition, the Trp64Arg polymorphism of *ADRβ3*gene is associated with insulin resistance in both diabetic (Burguete-Garcia et al. 2014) and non-diabetic obese patients (de Luis et al. 2007). The Arg64 variant is related to overweight, obesity and early onset of type 2 diabetes mellitus (Oeveren van-Dybicz et al. 2001), whereas the Trp64Trp polymorphism is associated with the highest effect of body mass reduction during low-energy diet and physical activity (de Luis et al. 2007).

Not only the genetic predisposition but also the unbalanced diet (intake of high fat and low amount of complex carbohydrate) can have an impact on glucose disorders (Post et al. 2012; Schulze et al. 2004; WHO 2003; Yoo et al. 2004; Grygiel-Gorniak et al. 2016). Recent studies have indicated that many vitamins of group B have beneficial influence on the glucose regulation (Agulló-Ortuño et al. 2002; Al-Maskari et al. 2012; Chow and Stone 1957; Gargari et al. 2011; Huang et al. 2013; Lazalde-Ramos et al. 2012; Sahin et al. 2013; Sasaki et al. 2012; Sudchada et al. 2012). Adequate vitamin B_12_ intake decreases the risk of hyperglycaemia (Chow and Stone 1957), whereas folate supplementation improves glycaemic control in diabetic patients (Gargari et al. 2011). Biotin beneficially influences the expression of the glucose transporter protein 4 (GLUT4) (Sahin et al. 2013; Sasaki et al. 2012). Pantothenic acid is synthesized during the activity of the vinin-1, which under fasting conditions modulates the glucose metabolism (Bell and Polonsky 2001). These data elucidate the important role of B vitamins in hyperglycaemia and underline their positive anti-diabetic effect (Lazalde-Ramos et al. 2012; Jianbo et al. 2011).

In view of the contentious association of *PPARγ2* and *ADRβ3* genes with hyperglycaemia, this study was planned to analyse selected polymorphisms of nutritional status, dietary habits and metabolic disorders in two groups of women who were diagnosed of impaired fasting glucose for the first time and never treated with hypoglycaemic medications.

## Material and methods

### Analysed group

The study included 271 postmenopausal women who have never been diagnosed and treated for hyperglycaemia or diabetes mellitus. They have been selected from 1431 subjects who were under the control of outpatient metabolic clinic. The postmenopausal period was assessed including the time (2 years from the last menorrhea) and hormonal criterion (serum follicle-stimulating hormone [FSH] concentration). Moreover, women with essential diseases such as non-treated thyroidal disorders, acute liver and renal diseases, neoplasm diagnosed during the past 5 years, acute infections and smoking, alcohol abuse or consumption of vitamins or mineral supplements were excluded from the study. All measurements (anthropometric and nutritional) were done using professional instruments, and a written informed consent was obtained from every woman enrolled in this study. This study was approved by the local Bioethics Committee of Poznan University of Medical Sciences (no. 792/09) and was performed according to the Helsinki Declaration.

### Blood pressure measurement

Blood pressure was measured by the auscultatory method with a mercury sphygmomanometer in the nondominant arm of the patient, after a 10-min rest in the upright seated position (between 7:00 am and 11:00 am after an overnight fast). Hypertension was defined as a systolic blood pressure (SBP) ≥ 140 mmHg and/or a diastolic blood pressure (DBP) ≥ 90 mmHg according to the recent guidelines of the European Society of Hypertension’s Working Group on Blood Pressure Monitoring (O’Brien et al. 2003).

### Anthropometric measurements

All the subjects were informed about the fasting and the anthropometric and biochemical analyses. Anthropometric measurements were done according to the current recommendations (Ness-Abramof and Apovian 2008), and the following components were included: body weight (kg; by a calibrated electronic scale to the nearest 0.1 kg, bare feet, in light clothing), body height (cm; by a vertical ruler to the nearest 0.5 cm), waist circumference (cm; to the nearest 0.1 cm, midway between the lower border of the ribs and the iliac crest at the widest portion) and hip circumference (cm; to the nearest 0.1 cm, at the widest diameter of the buttocks). Waist-to-hip ratio (WHR) and body mass index (BMI; body weight/ body height squared; kg/m^2^) were calculated according to World Health Organization (WHO) recommendations (WHO 2000). A high WHR > 0.85 in women indicated the visceral (abdominal) fat accumulation (WHO 2000). All anthropometric components were measured twice by study staff using a standardized protocol and averaged. The body fat mass and lean body mass (LBM) were assessed by the bioimpedance method using BODYSTAT 1500—a single-frequency device (50 kHz; Bodystat Ltd., Isle of Man, UK).

### Nutritional analysis

Nutritional intake was determined using an in-house developed 7-day estimated dietary record during normal diet (Charzewska 1998). The patients were trained to fill the questionnaires and qualified personnel verified the completed forms. The results of questionnaire studies were analysed based on the tables for the composition and nutrition value of food products (Kunachowicz et al. 1998). The intake of the analysed nutrients was compared with the recommended norms published by the National Institute of Food and Nutrition in Warsaw, Poland (Jarosz and Bulhak-Jachymczyk 2013). According to the data, the reduction of the intake of B vitamins was 20% (Jarosz and Bulhak-Jachymczyk 2013). The dietary intake of B vitamins was compared to the recommended norms of WHO statement and Polish recommendations, and the estimated average requirements (EAR) were as follows: 2 μg/day of vitamin B_12_ (EAR), 320 μg/day of folate (EAR), 5 mg/day adequate intake (AI) of pantothenic acid and 30 μg/day of biotin (AI) (Jarosz and Bulhak-Jachymczyk 2013; Food and Nutrition Board, Institute of Medicine, Natl Acad Board, Institute of Medicine 2000; WHO 2004). The dietary fibre intake was compared with the nutritional prophylaxis recommendation, which ranges from 27 to 40 g/day (Jarosz and Bulhak-Jachymczyk 2013).

### Biochemical analysis

Blood samples were taken between 7:00 am and 8:00 am after an overnight fast. Venous blood samples were collected in ethylenediaminetetraacetic acid-containing tubes, which were immediately centrifuged. Plasma glucose and lipid profiles (total cholesterol [TC], high-density lipoprotein [HDL] and triglycerides [TG]) were measured using enzymatic colorimetric assays (Cobas Integra 400 Plus; Roche Diagnostics, Indianapolis, IN). Low-density lipoprotein (LDL) was calculated from serum TC, TG and HDL according to the Friedewald equation (Friedewald et al. 1972). FSH serum levels were measured via specific chemiluminescence assays (Roche Diagnostics). Plasma insulin levels were determined by using an enzymatic immunoassay (Cobas Integra 400 Plus). Insulin resistance was estimated by homeostasis model assessment (HOMA) according to the formula: HOMA–insulin resistance (HOMA–IR) = fasting plasma glucose (mmol/L) × fasting insulin (mU/L) ∕ 22.5.

According to the European Society of Cardiology, analysed women were classified into two groups: normoglycaemic and hyperglycaemic based on venous fasting plasma glucose (FPG) (Rydén et al. 2013). An FPG ≤ 99 mg/dL (< 5.6 mmol/L) was normal, whereas FPG ≥ 100 ≤ 125 mg/dL (≥ 5.6 ≤ 6.9 mmol/L) was classified as impaired fasting glucose (Buysschaert and Bergman 2011; Rydén et al. 2013).

### Statistical analysis

The distribution of selected genotype and allele frequencies was analysed using Pearson’s chi-square test or Fisher’s exact test (for small frequencies) with calculated odds ratios (ORs). The expected frequencies of genotypes were determined by Hardy–Weinberg equilibrium deviation. The results of the study (continuous variables) were first verified according to the consistence with the normal distribution using the Shapiro–Wilk test. Generally, if the data passed the normality test, it was followed by parametric tests to compare the means (i.e. Student’s *t* test for two independent groups or one-way analysis of variance for more than two independent groups); otherwise, the nonparametric Mann–Whitney *U* test was used. The following variables were significantly different according to glucose level: BF, LBM, SBP, DBP, insulin, HDL, TG, intake of energy from fat and carbohydrates, vitamin B_12_, biotin and the Mann–Whitney *U* test. Then, the multivariate analysis by means of using classification trees was performed to find the continuous variables that are significantly associated with the studied polymorphisms and glucose. Quick, unbiased and efficient statistical tree (QUEST) algorithm was used for the analysis of classification trees. Two variables were found to be significantly associated with glucose in relation to polymorphisms: LBM and TG. In addition, ORs with 95% confidence interval were determined for hyperglycaemia related with the results of TG and LBM exceeding the norms. The statistical analysis was performed by using Statistica v. 12.0 (StatSoft, Inc.)

## Results

We did not find the differences (calculated by chi-square test) between the distribution of genotype and allele frequencies of the Pro12Ala, C1431T and Trp64Arg polymorphisms in two analysed groups of women, normo- and hyperglycaemic, and no significant deviation from the Hardy–Weinberg equilibrium was observed in our population (Table 1). Hyperglycaemic women (Table 2) have been found to be older than normoglycaemic women and were characterized with higher body mass, waist circumference, WHR, BMI, body fat amount, blood pressure (both diastolic and systolic), serum insulin, TG, calculated HOMA–IR, lower FSH level and higher intake of energy from fat. Moreover, they consume lower energy from the carbohydrates and lower amount of vitamins from group B (vitamin B_12_, biotin, pantothenic acid and folacin).

**Table 1:** Genotype and allele frequencies of the Pro12Ala and C1431/X *PPAR* gamma 2 and Trp64Arg of beta-adrenergic receptor gene polymorphisms according to normoglycaemic (glucose < 100 mg/dl) and hyperglycaemic state (glucose > 100 mg/dl). Data are *n* (%) for genotypes and *n* (frequency) for alleles

Body fat distribution	Normoglycaemic < 100 mg/dl *N* = 194		Hyperglycaemic ≥ 100 mg/dl *N* = 77		OR	95% CI	*p* value
	Observed *n* (%)	Expected (%)	Observed *n* (%)	Expected (%)			
Genotype (Pro12Ala)							
CC (Pro12Pro)	130 (67)	133 (68.6)	55 (71.4)	53 (68.8)	0.81	0.46–1.45	ns
CG (Pro12Ala)	57 (29.4)	54 (27.8)	19 (24.7)	22 (27.3)	1.23	0.69–2.19	ns
GG (Ala12Ala)	7 (3.6)	7 (3.6)	3 (3.9)	3 (3.9)	–	–	
Allele							
C	317 (81.70)	–	129 (83.77)	–			
G	71 (18.30)	–	25 (16.23)	–			
Genotype (C1431T)							
CC (C1431C)	143 (73.7)	157 (80.9)	55 (71.4)	63 (81.8)	1.21	0.62–2.02	ns
CT (C1431T)	47 (24.2)	34 (17.5)	19 (24.7)	13 (16.9)	0.89	0.49–1.61	ns
TT (T1431 T)	4 (2.1)	3 (1.5)	3 (3.39)	1 (1.3)	–		
Allele							
C	333 (85.82)	–	129 (83.77)	–			
T	55 (14.18)	–	25 (16.23)	–			
Genotype (Trp64Arg)							
TT (Trp64Trp)	159 (81.9)	142 (73.2)	61	56 (72.7)	1.19	0.62–2.31	ns
TC (Trp64Arg)	31 (16)	47 (24.2)	16	19 (24.7)	0.84	0.43–1.63	ns
CC (Arg64Arg)	4 (2.1)	5 (2.6)	–	2 (2.6)			
Allele							
T	349 (89.95)	–	138 (89.61)	–			
C	39 (10.05)	–	16 (10.39)	–

**Table 2:** Anthropometric, biochemical and nutritional characteristics of normo- and hyperglycaemic women

Analysed parameters	Normoglycaemic < 100 mg/dl *N* = 194	Hyperglycaemic ≥ 100 mg/dl *N* = 77	*p* value
Age (years)	58.73 ± 5.58	60.77 ± 4.89	0.0055
Body height (cm)	161.13 ± 6.02	160.47 ± 5.25	0.4048
Body mass (kg)	74.12 ± 16.52	82.02 ± 14.44	0.0003
WC (cm)	87.91 ± 14.66	96.61 ± 11.20	0.00001
WHR	0.83 ± 0.08	0.86 ± 0.06	0.0002
BMI (kg/m^2^)	28.49 ± 6.35	31.76 ± 5.63	0.0001
Body fat (%)	42.40 ± 6.63	46.16 ± 6.22	0.00001
LBM (%)	57.74 ± 6.51	53.77 ± 6.32	0.00001
Systolic pressure (mmHg)	137.06 ± 21.45	151.73 ± 21.86	0.00001
Diastolic pressure (mmHg)	86.08 ± 12.73	92.77 ± 14.50	0.0002
FSH (mIU/ml)	74.10 ± 20.77	59.89±	0.0001
Insulin (U/ml)	8.57 ± 7.32	12.49 ± 7.26	0.0001
Glucose (mg/dl)	90.28 ± 6.53	112.37 ± 13.77	0.00001
HOMA–IR [(mU/ml) × (mmol/l))]	1.93 ± 1.64	3.53± 2.33	0.00001
TC (mg/dl)	228.01 ± 39.96	238.10 ± 42.77	0.0673
HDL (mg/dl)	65.38 ± 14.94	60.50 ± 13.55	0.0135
TG (mg/dl)	105.56 ± 41.93	147.56 ± 67.58	0.0000
LDL (mg/dl)	141.55 ± 35.99	148.02 ± 38.36	0.1914
Energy (kcal)	2046.39 ± 552.40	2057.39 ± 575.11	0.8839
Protein (% energy)	16.27 ± 3.37	16.39 ± 3.09	0.8002
Fat (% energy)	33.58 ± 5.46	35.19 ± 4.70	0.0238
Carbohydrates (% energy)	51.03 ± 6.92	49.19 ± 6.05	0.0421
Dietary fibre (g)	22.75 ± 7.46	21.50 ± 7.01	0.2056
Vitamin B12 (μg)	2.97 ± 2.52	2.31 ± 1.65	0.0352
Biotyna (μg)	25.37 ± 11.54	22.36 ± 10.53	0.0479
Pantothenic acid (mg)	3.19 ± 1.17	2.81 ± 1.10	0.0164
Free folacin (μg)	115.82 ± 43.69	104.27 ± 39.39	0.0447
Total folacin (μg)	234.13 ± 86.69	211.79 ± 73.46	0.0472

The detailed data presented in Table 3 show the differences of analysed parameters between selected polymorphisms in normo- and hyperglycaemic women. Independent of the analysed polymorphism, hyperglycaemic women were characterized by lower LBM and serum HDL, lower intake of carbohydrates and B vitamins, higher body fat amount, SDP and DBP, serum insulin and TG, as well as dietary fat intake (in most cases, the differences were statistically significant). However, the analysis of Pro12Pro polymorphism has shown that the normoglycaemic women consumed higher energy from dietary fat (*p* < 0.0276) and lower energy from carbohydrates (*p* < 0.0480) compared to normoglycaemic women with Ala12/X polymorphism.

**Table 3:** Anthropometrical, biochemical and nutritional characteristics of postmenopausal women with polymorphisms of *PPAR* gamma 2 and *ADRβ3* genes

Analysed parameters	*X* ± SD	*X* ± SD	*p* value	*X* ± SD	*X* ± SD	*p* value
Polymorphism Pro12Ala	Polymorphism Ala12/X			Polymorphism Pro12Pro		
	Normoglycaemic, *n* = 64	Hyperglycaemic, *n* = 22		Normoglycaemic, *n* = 129	Hyperglycaemic, *n* = 55	
Body fat (%)	42.98 ± 6.97	45.73 ± 6.11	ns	42.11 ± 6.46	46.34 ± 6.31	0.0001
LBM (%)	57.01 ± 6.96	54.32 ± 6.14	ns	58.10 ± 6.28	53.55 ± 6.44	0.00001
Systolic pressure (mmHg)	135.80 ± 20.72	153.27 ± 25.71	0.0055	137.68 ± 21.84	151.11 ± 20.35	0.0002
Diastolic pressure (mmHg)	85.45 ± 12.29	94.59 ± 16.83	0.0377	86.38 ± 12.98	92.04 ± 13.56	0.0034
Insulin (mU/ml)	9.72 ± 11.37	12.05 ± 6.36	0.0043	8.00 ± 4.01	12.66 ± 7.63	0.00001
HDL (mg/dl)	64.96 ± 16.93	62.58 ± 10.88	ns	65.58 ± 13.92	59.67 ± 14.49	0.0084
TG (mg/dl)	109.20 ± 40.34	159.75 ± 89.96	0.0128	103.76 ± 42.73	142.68 ± 56.53	0.00001
Fat (% energy)	32.22 ± 4.45*	36.73 ± 5.11	0.0005	34.25 ± 5.80*	34.57 ± 4.43	ns
Carbohydrates (% energy)	52.72 ± 5.78**	47.25 ± 6.34	0.0002	50.19 ± 7.29*	49.97 ± 5.80	ns
VitaminB12 (μg)	3.04 ± 2.71	2.07 ± 1.15	0.0495	2.93 ± 2.43	2.41 ± 1.82	ns
Vitamin B7 (biotin) (μg)	25.93 ± 11.94	21.57 ± 9.30	ns	25.10 ± 11.37	22.67 ± 11.05	ns
Pantothenic acid (mg)	3.17 ± 1.08	2.70 ± 1.05	ns	3.20 ± 1.22	2.86 ± 1.13	0.0481
Polymorphism C1431T	Polymorphism T1431/X			Polymorphism C1431C		
	Normoglycaemic, *n* = 35	Hyperglycaemic, *n* = 16		Normoglycaemic, *n* = 158	Hyperglycaemic, *n* = 61	
Body fat (%)	43.56 ± 8.14	44.69 ± 7.23	ns	42.14 ± 6.24	46.55 ± 5.93	0.00001
LBM (%)	56.87 ± 7.78	55.31 ± 7.23	ns	57.93 ± 6.22	53.36 ± 6.06	0.00001
Systolic pressure (mmHg)	133.63 ± 21.67	156.06 ± 23.23	ns	137.82 ± 21.39	150.59 ± 21.54	0.00001
Diastolic pressure (mmHg)	84.74 ± 12.21	93.75 ± 14.22	ns	86.37 ± 12.86	92.51 ± 14.68	0.00001
Insulin (mU/ml)	8.02 ± 4.31	14.50 ± 7.29	0.0182	8.69 ± 7.83	11.96 ± 7.21	0.00001
HDL (mg/dl)	65.40 ± 14.96	64.24 ± 11.73	ns	65.37 ± 14.98	59.52 ± 13.91	0.0366
TG (mg/dl)	120.35 ± 56.10	130.16 ± 54.75	0.0250	102.30 ± 37.57	152.12 ± 70.24	0.00001
Fat (% energy)	33.81 ± 3.59	35.10 ± 4.87	ns	33.25 ± 5.54	35.55 ± 4.91	0.0148
Carbohydrates (% energy)	50.68 ± 3.71	49.54 ± 5.79	ns	51.36 ± 7.12	48.80 ± 6.49	0.0101
Vitamin B_12_ (μg)	3.52 ± 3.07	2.17 ± 0.96	0.0220	2.85 ± 2.38	2.35 ± 1.80	ns
Vitamin B_7_ (biotin) (μg)	27.67 ± 12.48	21.47 ± 8.63	0.0493	24.87 ± 11.30	22.59 ± 11.02	ns
Pantothenic acid (mg)	3.40 ± 1.3	3.00 ± 1.15	0.0319	3.14 ± 1.14	2.77 ± 1.09	ns
Polymorphism Trp64Arg	Arg64/X			Trp64/Trp		
	Normoglycaemic, *n* = 35	Hyperglycaemic, *n* = 16		Normoglycaemic, *n* = 158	Hyperglycaemic, *n* = 61	
Body fat (%)	43.56 ± 8.14	44.69 ± 7.23	ns	42.14 ± 6.24	46.55 ± 5.93	0.00001
LBM (%)	56.87 ± 7.78	55.31 ± 7.23	ns	57.93 ± 6.22	53.36 ± 6.06	0.00001
Systolic pressure (mmHg)	133.63 ± 21.67	156.06 ± 23.23	0.0033	137.82 ± 21.39	150.59 ± 21.54	0.0003
Diastolic pressure (mmHg)	84.74 ± 12.21	93.75 ± 14.22	0.0217	86.37 ± 12.86	92.51 ± 14.68	0.0031
Insulin (mU/ml)	8.02 ± 4.31	14.50 ± 7.29	0.0014	8.69 ± 7.83	11.96 ± 7.21	0.00001
HDL (mg/dl)	65.40 ± 14.96	64.24 ± 11.73	ns	65.37 ± 14.98	59.52 ± 13.91	0.0101
TG (mg/dl)	120.35 ± 56.10	130.16 ± 54.75	ns	102.30 ± 37.57	152.12 ± 70.24	0.0000
Fat (% energy)	33.81 ± 3.59	35.10 ± 4.87	ns	33.25 ± 5.54	35.55 ± 4.91	0.0008
Carbohydrates (% energy)	50.68 ± 3.71	49.54 ± 5.79	ns	51.36 ± 7.12	48.80 ± 6.49	0.0035
Vitamin B_12_ (μg)	3.52 ± 3.07	2.17 ± 0.96	ns	2.85 ± 2.38	2.35 ± 1.80	ns
Vitamin B_7_ (biotin) (μg)	27.67 ± 12.48	21.47 ± 8.63	ns	24.87 ± 11.30	22.59 ± 11.02	ns
Pantothenic acid (mg)	3.40 ± 1.31	3.00 ± 1.15	ns	3.14 ± 1.14	2.77 ± 1.09	0.0175

*LBM* lean body mass

*p < 0.0276; **p < 0.0480 *p* values between analysed polymorphisms

Independent of the glycaemic state, women characterized by Pro12Pro polymorphism and higher amount of LBM (> 58% of body mass) had higher chance of normal glucose level compared to women with lower LBM (*p* = 0.0007; 74.2% of analysed subjects were normoglycaemic) (Table 4). In addition, women with Trp64Trp polymorphism and low level of TG had higher chance of normoglycaemic condition than women with hypertriglyceridaemia (TG ≥ 150 mg/dL, *p* = 0.0002).

**Table 4:** Analysis of the risk of hyperglycaemia in analysed groups

Analysed polymorphism	Analysed groups	*n*	%	*n*	%
Pro12Pro		LBM > 58%		LBM ≤ 58	
	Normoglycaemic	184	74.2	17	51.5
	Hyperglycaemic	64	25.8	16	48.5
	Sum	248	100	33	100
		OR = 3.44	(1.69–7.01)	OR = 0.29	(0.14–0.59)
		*p* = 0.0007			
Trp64Trp		TG ≤ 150 mg/dl		TG > 150 mg/dl	
	Normoglycaemic	140	80.5	19	41.3
	Hyperglycaemic	40	19.5	21	58.7
	Sum	180	100	46	100
		OR = 3.87	(1.90–7.89)	OR = 0.26	(0.13–0.53)
		*p* = 0.0002			

## Discussion

The hyperglycaemic state was first time diagnosed in 28.4% of analysed women (*n* = 77; average venous FPG was 112.37 ± 13.77 mg/dL). Analysed women were never treated with diabetic diet or anti-diabetic medications. Hyperglycaemic women were older than normoglycaemic women, which may reflect the fact that the risk of hyperglycaemia and diabetes mellitus increased with age (Halter et al. 2014). Moreover, they were viscerally obese (BMI > 30 kg/m^2^, WHR ≥ 0.85) (WHO 2000). Ageing and, in particular, menopause transition are associated with increased prevalence of abdominal fat deposition and metabolic disorders (Carr and Brunzell 2004). In this study, hyperglycaemic women had worse metabolic profile (higher serum TG, TC and calculated HOMA–IR), which increased the risk of the diabetes mellitus and cardiovascular disease (CVD) development in the future (Carr and Brunzell 2004). Their blood pressures were elevated, and the DBP was also increased in normoglycaemic overweight women.

Even the energy intake was found to be within the recommended level, and the daily food ratios were improperly balanced and contained excess of fat along with insufficient consumption of carbohydrates. The high percentage of energy from fat is epidemiologically alerting, and the amount of this nutrient should not exceed 30% of daily energy intake in general population; however, if the BMI is > 25 kg/m^2^ (like in this study), the value should not be higher than 25% of energy from this component (Jarosz and Bulhak-Jachymczyk 2013; WHO 2003). The low percentage of energy from carbohydrates did not achieve the recommended amount of 55–75% of energy intake (including minimum 50–70% polysaccharides) (WHO 2003). In addition, low intake of dietary fibre is the risk of hyperglycaemic complications (WHO 2003). It was proved that the diet composed of high complex carbohydrates and dietary fibre is beneficial in the reduction of body mass and diabetes mellitus development (Post et al. 2012)**.**

Besides the basic dietary components, adequate consumption of B vitamins also has beneficial influence on the glycaemic state. Unfortunately, in this study, only the intake of vitamin B_12_ was proper, whereas the intake of other vitamins from group B (folate, biotin and pantothenic acid) was lower than the recommended level (Jarosz and Bulhak-Jachymczyk 2013; Food and Nutrition Board, Institute of Medicine, Natl Acad Board, Institute of Medicine 2000; WHO 2004). Considering the role of B vitamins in carbohydrate metabolism, their low intake in the analysed group may increase the risk of hyperglycaemia. Cobalamin not only has anti-oxidative properties (Al-Maskari et al. 2012; Huang et al. 2013) but also participates in the utilisation of carbohydrates (Chow and Stone 1957), whereas folate modulates the glucose metabolism (Lazalde-Ramos et al. 2012). Folate supplementation decreases glycosylated haemoglobin, fasting blood glucose, insulin and homocysteine in type 2 diabetes mellitus patients (Gargari et al. 2011). The AI of biotin (which increases GLUT4 and raises insulin sensitivity in skeletal muscle) (Sahin et al. 2013; Sasaki et al. 2012) and proper pantothenic acid consumption (Chen et al. 2014) cause an anti-diabetic effect.

In Table 3, we have presented the detailed analysis of body components, metabolic parameters and nutritional factors in the discussed groups of women. Most differences between normo- and hyperglycaemic women were observed within the same polymorphism. However, normoglycaemic women with *Ala* allele consumed lower energy from fat and higher energy from carbohydrates than normoglycaemic women with Pro12Pro polymorphism. Many studies showed that Ala12 polymorphism is related to insulin sensitivity (Douglas et al. 2001; Ek et al. 2001), and a meta-analysis of Altshuler et al. has revealed a significant reduction of risk of diabetes of 21% in subjects with *Ala* allele (Lazalde-Ramos et al. 2012).

Women with Pro12Pro polymorphism (normo- and hyperglycaemic) and higher LBM (> 58% of body mass) have bigger chance of normal glucose level compared to subjects with lower LBM (Table 4). Thus, besides the predisposition of Pro12Pro to hyperglycaemia and diabetes mellitus development (Bell and Polonsky 2001; Jaziri et al. 2006), the high LBM (which could be caused by increased physical activity) can prevent the glycaemic complication. Many studies have confirmed that the subjects with high muscle mass and regular physical activity had lower risk of diabetes mellitus (Lee et al. 2010; Shishikura et al. 2014). In addition, women with Trp64Trp polymorphism and low level of TG have higher chance of normoglycaemic state than women with hypertriglyceridaemia. In the reference data, the Trp64Arg allele is associated with lower risk of insulin resistance in diabetic (Burguete-Garcia et al. 2014) and non-diabetic obese patients (de Luis et al. 2007), whereas the Arg64 variant is reported to be related to overweight, obesity and early onset of type 2 diabetes mellitus (Oeveren van-Dybicz et al. 2001; Zhan and Ho 2005). Moreover, high TG level is one of the components of metabolic syndrome and coexists with hyperglycaemia (Alberti et al. 2009; Weitgasser et al. 2004). In this study, women with Trp64Trp polymorphism and normal TG level were mainly predicted by normal glucose level, which indicates the beneficial influence of this polymorphism on metabolic parameters.

## Conclusion

The risk factors of hyperglycaemia in postmenopausal women include high body mass and body fat, visceral distribution of fat, low LBM and improperly balanced diet (high fat intake and lower amount of complex carbohydrates and B vitamins). The presence of Pro12Pro genotype is related to higher risk of diabetes mellitus and is associated with worse dietary habits; however, this risk is reduced in subjects with higher muscle mass. Moreover, the normal TG level in women withTrp64Trp polymorphism predicts normoglycaemia. Thus, the predisposition to hyperglycaemia is multifactorial; not only the genetic background but also dietary habits (fat overnutrition and intake of low B vitamins) influence the risk of high glucose level and might precede diabetes mellitus development.
